# Global Trends in Norovirus Genotype Distribution among Children with Acute Gastroenteritis

**DOI:** 10.3201/eid2705.204756

**Published:** 2021-05

**Authors:** Jennifer L. Cannon, Joseph Bonifacio, Filemon Bucardo, Javier Buesa, Leesa Bruggink, Martin Chi-Wai Chan, Tulio M. Fumian, Sidhartha Giri, Mark D. Gonzalez, Joanne Hewitt, Jih-Hui Lin, Janet Mans, Christian Muñoz, Chao-Yang Pan, Xiao-Li Pang, Corinna Pietsch, Mustafiz Rahman, Naomi Sakon, Rangaraj Selvarangan, Hannah Browne, Leslie Barclay, Jan Vinjé

**Affiliations:** National Foundation for the Centers for Disease Control and Prevention, Inc., Atlanta, Georgia, USA (J.L. Cannon, H. Browne);; Research Institute for Tropical Medicine, Manila, the Philippines (J. Bonifacio);; National Autonomous University of Nicaragua, León, Nicaragua (F. Bucardo);; University of Valencia, Valencia, Spain (J. Buesa);; Peter Doherty Institute for Infection and Immunity, Melbourne, Victoria, Australia (L. Bruggink);; Chinese University of Hong Kong, Hong Kong, China (M.C.-W. Chan);; Institute Oswaldo Cruz, Rio de Janeiro, Brazil (T.M. Fumian);; Christian Medical College, Vellore, India (S. Giri);; Children’s Healthcare of Atlanta, Atlanta (M.D. Gonzalez);; Institute of Environmental Science and Research, Porirua, New Zealand (J. Hewitt);; Taiwan Centers for Disease Control, Taipei, Taiwan (J.-H. Lin);; University of Pretoria, Pretoria, South Africa (J. Mans);; University of Antofagasta, Antofagasta, Chile (C. Muñoz);; California Department of Public Health, Richmond, California, USA (C.-Y. Pan);; Alberta Precision Laboratory, Edmonton, Alberta, Canada (X.-L. Pang);; Leipzig University Hospital, Leipzig, Germany (C. Pietsch);; International Centre for Diarrhoeal Disease Research, Bangladesh, Dhaka, Bangladesh (M. Rahman);; Osaka Institute of Public Health, Osaka, Japan (N. Sakon);; Children’s Mercy Hospitals and Clinics, Kansas City, Missouri, USA (R. Selvarangan);; Centers for Disease Control and Prevention, Atlanta (L. Barclay, J. Vinjé)

**Keywords:** norovirus, children, gastroenteritis, genotypes, vaccines, capsids, polymerase, surveillance, NoroSurv, dual typing, acute gastroenteritis, P-types, viruses, enteric infections, food safety

## Abstract

Noroviruses are a leading cause of acute gastroenteritis (AGE) among adults and children worldwide. NoroSurv is a global network for norovirus strain surveillance among children <5 years of age with AGE. Participants in 16 countries across 6 continents used standardized protocols for dual typing (genotype and polymerase type) and uploaded 1,325 dual-typed sequences to the NoroSurv web portal during 2016–2020. More than 50% of submitted sequences were GII.4 Sydney[P16] or GII.4 Sydney[P31] strains. Other common strains included GII.2[P16], GII.3[P12], GII.6[P7], and GI.3[P3] viruses. In total, 22 genotypes and 36 dual types, including GII.3 and GII.20 viruses with rarely reported polymerase types, were detected, reflecting high strain diversity. Surveillance data captured in NoroSurv enables the monitoring of trends in norovirus strains associated childhood AGE throughout the world on a near real-time basis.

Globally, noroviruses are associated with ≈20% of acute gastroenteritis (AGE) cases, causing an estimated 685 million episodes and 210,000 deaths each year (*1*,*2*). By 2 years of age, children have probably had >1 norovirus infection (*3*–*5*). Children in this age group are at risk for severe illness, prolonged symptoms, and infection by multiple strains (*3*–*5*). Sporadic illnesses among children might contribute to community transmission and outbreaks among all age groups (*6*). In countries with successful rotavirus vaccination campaigns, norovirus is now the most common cause of pediatric AGE requiring medical attention (*7*–*9*). As of January 2021, vaccines for norovirus are in clinical trials (phase I and II) and developmental stages (*10*). However, their design is challenging because of the high genetic diversity of noroviruses and incomplete understanding of cross-protective immunity (*11*). If candidate vaccines are successful at blocking onward transmission events, norovirus vaccination will benefit children and unvaccinated persons across all age groups (*12*).

Norovirus classification is based on amino acid diversity of the major capsid protein (encoded by open reading frame [ORF] 2), which is also the primary neutralization site for antibodies produced after norovirus infection or vaccination (*13*–*15*). Noroviruses are classified into 10 genogroups, GI–GX, and >48 genotypes: 9 genotypes in the GI genogroup, 26 in GII, 3 in GIII, 2 in GIV, 2 in GV, 2 in GVI, 1 in GVII, 1 in GVIII, 1 in GIX, and 1 in GX (*16*). ORF1 encodes the viral nonstructural proteins including the polymerase, which is classified into >60 polymerase types (P-types) (*16*). Much about the evolutionary role of recombination among noroviruses, which occurs primarily at the ORF1/ORF2 junction, remains unknown (*17*–*19*). Norovirus classification was recently updated to include typing of the polymerase region (*16*). This dual typing strategy considers the genotype encoding the major capsid protein and the P-type encoding the polymerase region (*16*). A short genomic region spanning the 3′ end of the polymerase gene through the 5′ end of the capsid gene is the basis for sequence-based dual typing (*20*).

Genogroup II genotype 4 (GII.4) viruses have been the most frequently detected noroviruses globally since the mid-1990s, before which GII.3 viruses were dominant (*13*,*21*,*22*). New GII.4 variants regularly emerge and spread across the globe and often contribute to increased illness and death, especially in healthcare settings (*23*–*25*). During 2002–2012, new GII.4 variants with antigenically distinct capsid epitopes, which enable the viruses to escape neutralizing antibodies, emerged and replaced previous variants every 2–3 years (*15*). These changes indicate that norovirus vaccines might need to be updated regularly. Despite recent recombination events resulting in the global spread of GII.4 Sydney viruses with a novel P16 polymerase, no new variant causing widespread infections has emerged since 2012 (*20*,*26*,*27*). Although GII.4 strains are the most common strains detected among all age groups, non-GII.4 strains, such as GII.2, GII.3, and GII.6 viruses, are common causes of sporadic cases and illness in young children (*6*,*28*–*32*). Rare strains (*4*) and GII.4 variants can circulate, especially among children, for years before spreading globally among all age groups (*33*,*34*). Consequently, children might be an important reservoir for emerging norovirus strains against which little or no population immunity exists.

NoroSurv (https://www.norosurv.org), which is maintained by the Centers for Disease Control and Prevention (Atlanta, Georgia, USA), is a global pediatric norovirus strain surveillance network for children <5 years of age with medically attended AGE and can only be accessed by registered NoroSurv laboratories. Surveillance of norovirus strains infecting children is crucial for monitoring the emergence of new or rare strains and for developing vaccines that protect against the most common strains.

## Methods

### NoroSurv

All but 2 participating hospitals and medical centers collected norovirus–positive stool samples from children with AGE; 2 sites in Nicaragua and Australia obtained only samples from symptomatic children in community-based studies. Staff at hospitals, medical centers, universities, and reference laboratories processed and typed the samples. Each laboratory uploaded norovirus sequences; patient demographic data (e.g., deidentified patient age and sex); and information on sample type, collection date, and setting to the password-protected NoroSurv web portal. All laboratories used a standardized protocol for norovirus dual typing that comprised screening by genotype-specific real-time reverse transcription PCR (RT-PCR), conventional RT-PCR, and Sanger sequencing of RT-PCR products (*20*). Raw DNA chromatogram files or nucleotide sequences were automatically typed by NoroSurv using the most recent reference sequences and classification for noroviruses (*16*). Ethics approval for the New Zealand component of this study was granted by the Health and Disability Ethics Committee, New Zealand (approval no. 19/CEN/96).

### Data Analysis

We analyzed NoroSurv data associated with samples collected during September 1, 2016–August 31, 2020. We excluded samples from children >5 years of age, from asymptomatic patients, or that had missing or low-quality dual typing information. We downloaded sequences and associated data from NoroSurv; we then aggregated, cleaned, analyzed, and visualized the data using R software (The R Project, https://www.r-project.org). After downloading the sequences from NoroSurv as fasta files, we checked the quality of the submitted sequences using Bioconductor (http://bioconductor.org) packages in R. When discrepancies existed between the manually entered and autotyped information, we conducted phylogenetic analysis to confirm the correct type; we updated NoroSurv records accordingly.

## Results

**Figure 1:**
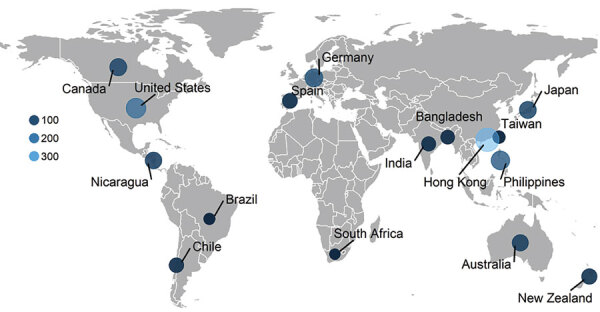
Countries participating in NoroSurv, December 2016–August 2020. Shades of blue and size of circles indicate the number of genetic sequences included from each country.

A total of 1,325 dual-typed norovirus sequences collected during September 2016–August 2020 from children <5 years of age with AGE were submitted to NoroSurv. Sequences were received from 19 sites in 16 countries in Africa (South Africa, n = 13), Asia (Bangladesh, n = 32; Hong Kong, China, n = 326; India, n = 36; Japan, n = 89; the Philippines, n = 132; and Taiwan, n = 19), Oceania (Australia, n = 71; New Zealand, n = 54), Europe (Germany, n = 111 and Spain, n = 44), North and Central America (Canada, n = 90; Nicaragua, n = 78; and the United States, n = 173), and South America (Brazil, n = 14 and Chile, n = 43) (Figure 1). Each country submitted a median of 63 sequences (range 13–326); 48% of sequences were from countries in Asia. We excluded 62 of the 1,387 NoroSurv sequences: 11 that could not be typed because of poor sequence quality or missing fasta files; 7 with sample collection dates before September 1, 2016; 31 from children >5 years of age; and 13 from asymptomatic children.

**Figure 2:**
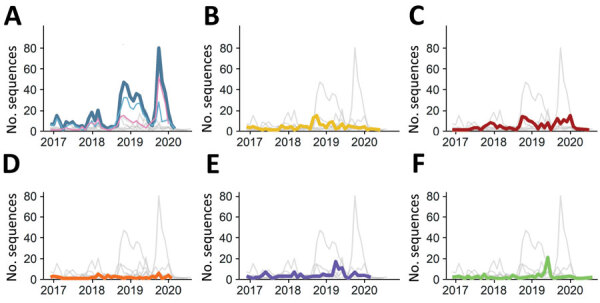
Global genotype distribution of norovirus sequences catalogued in NoroSurv during September 2016–August 2020. A) Dark blue line indicates all GII.4 Sydney viruses; light blue indicates GII.4 Sydney[P16] and pink indicates GII.4 Sydney[P31]); B) yellow indicates GII.2 viruses; C) red indicates GII.3 viruses; D) orange indicates GII.6 viruses; E) purple indicates other GII viruses; F) green indicates GI viruses. Gray lines overlay the distributions of other pictured genotypes to enable comparisons.

To compare genotype distribution over time, we defined seasons as September 1–August 31; these periods reflected the seasonality reported for noroviruses, with peak cases often occurring during the cooler months: October–March in the Northern Hemisphere and April–September in the Southern Hemisphere (*35*). During the pilot phase (September 1, 2016–August 31, 2018), a total of 382 sequences were submitted (144 in 2016–2017 and 238 in 2017–2018). During the first 2 official years of NoroSurv, 600 sequences were submitted in the 2018–2019 season and 343 in 2019–2020 season. The number of submissions peaked between the months of October and May (Figure 2), coinciding with cooler months in the Northern Hemisphere. However, only 15% (195/1,325) of sequences were submitted by Southern Hemisphere countries; for this reason, analyzing trends in the Southern Hemisphere was difficult. Many sample collection sites in the Philippines were equatorial and had norovirus cases year-round. The number of submitted sequences declined in 2020, coinciding with the emergence of the coronavirus disease pandemic (Figure 2).

**Figure 3:**
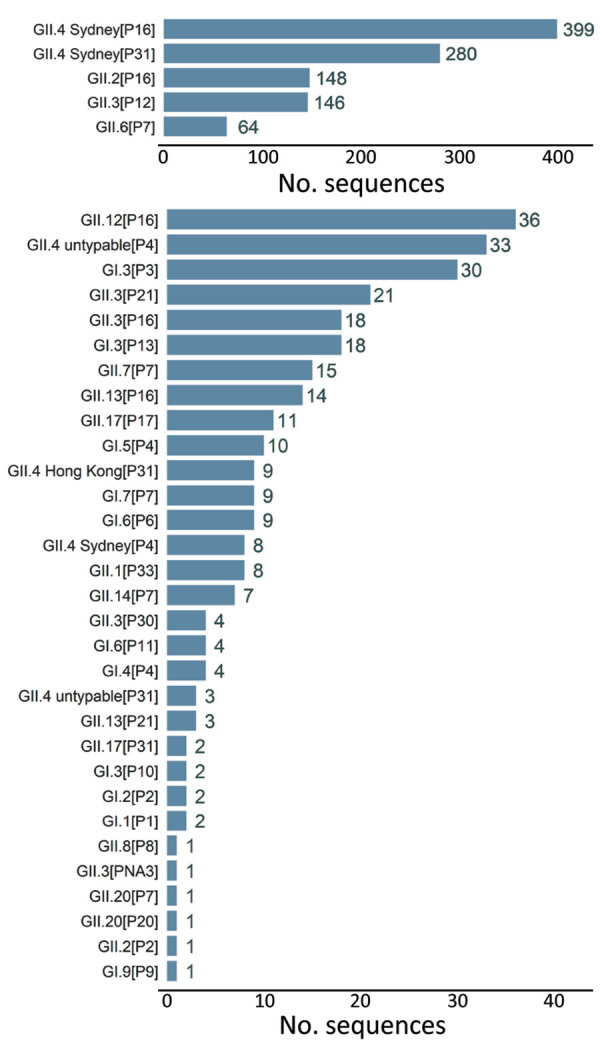
Distribution of dual typed sequences in NoroSurv, 2016–2020. Numbers to the right of bars indicate the number of sequences detected for each dual type.

Throughout the study period, GII.4 Sydney was the most common genotype on all 6 continents and was detected in 52% (687/1,325) of sequences, peaking at 62% (213/343) in 2019–2020 (Figure 2; Appendix Table 1). The GII.3 (190; 14%), GII.2 (149; 11%), and GII.6 (64; 5%) genotypes comprised 30% of sequences (Figure 2; Appendix Table 1). GI.3 was the most frequently detected GI genotype, accounting for 55% (50/91) of all GI viruses and 4% of all NoroSurv sequences. The remaining 14% (185/1,325) of sequences were composed of 17 other genotypes: GI.1, GI.2, GI.4, GI.5, GI.6, GI.7, GI.9, GII.1, GII.4 Hong Kong, GII.4 untypable, GII.7, GII.8, GII.12, GII.13, GII.14, GII.17, and GII.20 (Appendix Table 1). We detected 687 GII.4 Sydney viruses associated with 3 P-types: P16 (399; 58%), P31 (280; 41%), and P4 (8; 1%). The proportions of each genotype varied by year (Figure 2; Appendix Table 1) and country (Appendix Tables 2–17). The most common P-type among the 190 detected GII.3 viruses was P12 (146; 77%) (Figure 3; Appendix Table 1). We detected 149 GII.2 viruses, most (148; 99%) of which were P16. All 64 GII.6 viruses were P7 (Figure 3; Appendix Table 1).

The 5 most frequently detected dual types were GII.4 Sydney[P16], GII.4 Sydney[P31], GII.2[P16], GII.3[P12], and GII.6[P7]. In total, 22% (288/1,325) of sequences were composed of 31 other dual types, each accounting for <5% of all sequences (Figure 3; Appendix Table 1). The 10 most frequently detected dual types included GII.12[P16], GII.4 untypeable[P4], GI.[P3], GII.3[P21], and GII.3[P16] (Figure 3). We found that the 23 GII.4 untypeable[P4] viruses detected in Chile, 4 in the United States, 3 in Australia, 1 in Germany, 1 in New Zealand, and 4 in Spain (1 P4 and 3 P31) formed a GII.4 Sydney subclade. This subclade exceeded the >2% designated cutoff for percent nucleotide differences between these strains and the closest GII.4 Sydney reference sequence (GenBank accession no. KX354134, mean nucleotide percent difference = 2.2%, SD = 0.3%). Several genotypes were associated with >2 P-types. For example, GII.3 viruses were associated with P12, P21, P16, P30, and PNA3; GI.3 viruses were associated with P3, P13, and P10; and GII.13 viruses were associated with P16 and P21 (Figure 3; Appendix Table 1). We also detected dual types rarely reported in literature, including GII.3[PNA3] in South Africa; GII.20[P20] and GII.20[P7] in New Zealand; and GII.3[P30] in Hong Kong, Canada, and Spain (Appendix Tables 5, 8, 11, 14, 15).

During the 2019–2020 season, 65% (138/213) of GII.4 Sydney viruses had a P31 polymerase, compared with only 28% (89/314) in the previous season (Figure 2; Appendix Table 1). This dual type was most common (115; 81%) in Hong Kong (Appendix Table 8). In total, sites in Hong Kong submitted 25% (326/1,325) of all NoroSurv sequences, including 46% (159/343) in 2019–2020. In Japan, South Africa, and Taiwan, GII.4 Sydney[P31] viruses were also more common than GII.4 Sydney[P16] viruses (Appendix Tables 10, 14, 16). In the 12 remaining countries, GII.4 Sydney[P16] viruses were either more than or as common as GII.4 Sydney[P31] viruses (Appendix Tables 2–7, 9, 11–13, 15, 17).

GII.4 Sydney viruses were the most common virus in all but 3 countries: GII.3[P12] viruses were most common in New Zealand (26/54; 48%) and Taiwan (7/19; 37%) and GII.4 untypeable[P4] viruses were most common in Chile (23/43; 53%) (Figure 3; Appendix Tables 6, 11, 16). Norovirus strain diversity was high in many countries, with >10 strains detected in 7 countries: 18 each in the Philippines and the United States, 16 in Spain, 15 in Germany, 13 in Hong Kong, and 12 each in Australia and New Zealand (Appendix Tables 2, 7, 8, 11, 13, 15, 17).

## Discussion

We used NoroSurv data to monitor global trends in norovirus genotypes causing sporadic AGE in children <5 years of age. These children would probably benefit most from norovirus vaccines and are a critical group for evaluating future vaccine effectiveness. Although the number of sequences submitted from different countries varied during 2016–2020 on NoroSurv, the overall surveillance from 16 countries across 6 continents identified common genotypes around the world. Standardized protocols for dual typing across all NoroSurv sites enabled global comparisons, surveillance, and detection of recombinant strains.

During 2016–2020, NoroSurv documented 22 genotypes of norovirus causing illness in young children. GII.4 Sydney viruses, which globally are the most common among all age groups (*13*,*26*), comprised >50% of all NoroSurv sequences. GII.2, GII.3, and GII.6 viruses, which are leading causes of childhood norovirus infections but less common among adults, were also frequently detected (*6*,*28*,*30*–*32*). One study found that among children with sporadic AGE, GII.6 viruses were second most common after GII.4; however, GII.13 viruses were the second most common cause of noroviruses outbreaks in adults (*6*). Although ≈5% of reported norovirus outbreaks in the United States are caused by GII.3 viruses (*17*,*20*), we found they comprised nearly 23% of sporadic cases among children. Thus, GII.2, GII.3, and GII.6 viruses appear to be major causes of AGE in children but might be less transmissible to adults. This lack of transmissibility might be caused by virus-specific properties or long-term immunity in adults after childhood infection. Norovirus vaccines in development focus on the major capsid protein, which is also the genomic region used for genotyping (*14*,*16*). Vaccine candidates should protect against a broad diversity of genotypes and be easily adapted to emerging genotypes or GII.4 variants. Noroviruses contribute substantially to the prevalence of diarrheal disease among children (*1*), causing more severe illness and death in resource-limited countries (*30*,*36*). Childhood vaccination might reduce norovirus prevalence among children. If vaccination prevents transmission, then it also might reduce infections among all age groups (*6*,*12*).

GII.4 Sydney viruses, primarily associated with P31 and P16 polymerases, were responsible for most norovirus cases during 2012–2019 (*33*,*34*). Recombination at the ORF1/ORF2 junction is a common occurrence among noroviruses and contributes to norovirus evolution, although the exact mechanism is poorly understood (*17*–*19*). Acquisition of a novel P16 polymerase did not result in emergence of a novel GII.4 variant or substantial changes to the antigenic region of the capsid (*17*,*37*,*38*). However, changes to the polymerase or other nonstructural proteins might have increased the replicative or transmission fitness of GII.4 viruses (*17*,*18*,*26*).

Overall, 36 dual types were detected in NoroSurv and several genotypes were associated with >1 P-type. GII.3 viruses were primarily associated with P12, but many had P21, P16, and the rare P30 and PNA3 polymerases, indicating a high propensity for recombination among GII.3 strains. Other rarely detected strains included GII.20[P20] and GII.20[P7]. Several rare and novel norovirus genotypes have been detected only in children (*4*), suggesting differences in children’s and adults’ susceptibility to certain strains. We identified a subcluster of GII.4 Sydney (GII.4 untypable) viruses in 6 countries spanning 4 continents during 2017–2019. Complete ORF2 sequences for this strain are needed to analyze possible changes in the antigenic region of the capsid, which could enable viruses to escape antibody neutralization. If such changes exist, or if strains within this subcluster continue to evolve and spread globally, a new GII.4 variant could emerge. A recent study reported that GII.4 variants can begin to circulate, especially among children, for up to 9 years before emerging globally (*33*). Low-level circulation enables accumulation of mutations and emergence of new strains (*18*,*38*) and access to niches in the host environment, thereby promoting spread (*33*); thus, children might be a reservoir for the recombination and evolution of noroviruses. This concern highlights the necessity of norovirus surveillance among children.

NoroSurv complements NoroNet (*34*), a well-established global network for norovirus surveillance that has illuminated global trends in norovirus strain diversity, recombination, and evolution, including tracking the emergence of novel GII.4 variants. NoroSurv sequences are derived from sporadic cases among children, whereas NoroNet includes sequences from outbreaks and sporadic cases in adults and children. NoroSurv requires standardized protocols for dual typing (*20*) across all sites to ensure global comparability. However, NoroNet, which was established in 1999, has a much longer history than NoroSurv. Because the importance of dual typing was not well recognized at the time NoroNet was established, many of its sequences are derived from either the polymerase or capsid genes, but not both. In addition, the NoroSurv web portal incorporates a unique automatic typing tool and an internal dashboard of all data by location. In 2021, we plan to make the dashboard publicly available for near real-time data on global trends in sporadic norovirus infections in children.

NoroSurv is a passive surveillance system comprised of voluntary submissions from participating laboratories. As a result, its data do not necessarily correlate with national surveillance records. Furthermore, the number of sequences submitted from each country varies; this number depends in part on the availability of resources such as time and laboratory capacity. Low-income countries are currently underrepresented in NoroSurv, as are countries in Africa and Central America. However, a recent review of norovirus genotypes detected in 8 low-income and 21 low-to-middle income countries showed that GII.4 viruses were the most common genotype, with substantial proportions of GII.3 and GII.6 viruses; in addition, GI.3 viruses were the most commonly detected GI viruses (*30*). Trends in the genotype distribution of noroviruses in these countries resembled the global trends illuminated in NoroSurv. In future years, NoroSurv aims to expand of the number of countries, sites, and submissions.

The 2019–20 norovirus season coincided with the emergence of the coronavirus disease pandemic, which has limited the capacity and resources for norovirus surveillance. In addition, it is unknown whether the global lockdowns, including school and daycare closures; physical distancing; and heightened hygiene awareness and practices such as handwashing, disinfection, and wearing of face masks (*39*), will reduce norovirus transmission among children. When settings prone to norovirus outbreaks (e.g., childcare facilities and schools) return to prepandemic capacities, norovirus cases might increase, especially if the use of alcohol-based hand sanitizers, which have limited efficacy against noroviruses (*40*), are substituted for handwashing in these settings. Although submissions to NoroSurv declined during February–August 2020, users might upload sequences retrospectively. As a result, data for the 2019–2020 season might not fully reflect global trends.

NoroSurv enables the near real-time detection of global norovirus genotype trends and diversity among children <5 years of age with AGE. Our findings support previous research indicating that although some overlap exists between the genotypes detected in children and adults, genotypes such as GII.2, GII.3, and GII.6 are more common among children. Childhood norovirus vaccination will probably reduce the prevalence of norovirus associated AGE among children and interrupt community transmission among all age groups (*12*). As such, researchers should ensure that candidate vaccines are protective against strains commonly seen in children or produce sufficient cross-protective immunity against those strains. Surveillance of rare genotypes, recombinant strains, and potentially new GII.4 variants can better predict the emergence of new strains, guiding potentially updated vaccine formulation. Sequencing larger regions of the genome, particularly the major capsid gene, can help identify antigenic changes that might enable the virus to escape antibody neutralization, which provides important information for predicting strain emergence and updating vaccine formulations. The continued expansion of the NoroSurv network to include countries with geographic and economic diversity will enhance our understanding of norovirus infections among children worldwide. NoroSurv surveillance will inform efforts to develop and adapt norovirus vaccine candidates; it will also aid in the evaluation of future vaccine efficacy by documenting baseline global strain diversity of noroviruses in children.

Appendix Further data on global trends in norovirus genotype distribution among children with acute gastroenteritis.
