# Supplementary material for: Global Trends in Norovirus Genotype Distribution among Children with Acute Gastroenteritis
Source: Emerg Infect Dis. 2021 May;27(5):1438–45. doi: 10.3201/eid2705.204756 (PMC8084493; doi:10.3201/eid2705.204756)
Supplement: Appendix — Further data on global trends in norovirus genotype distribution among children with acute gastroenteritis. [file 20-4756-Techapp-s1.pdf]

Article DOI: https://doi.org/10.3201/eid2705.204756

Global Trends in Norovirus Genotype Distribution among Children with Acute Gastroenteritis

Appendix

Appendix Table 1. All dual-typed norovirus sequences in a global study of norovirus genotype distribution among children with acute gastroenteritis, 2016–2020

	Season, no. (%)				Total
Genotype, P-type	2016–2017	2017–2018	2018–2019	2019–2020	no. (%)
GI.1	1 (1)	0	0	1 (<1)	2 (<1)
P1	1 (1)	0	0	1 (<1)	2 (<1)
GI.2	0	2 (1)	0	0	2 (<1)
P2	0	2 (1)	0	0	2 (<1)
GI.3	4 (3)	10 (4)	30 (5)	6 (2)	50 (4)
P3	2 (1)	4 (2)	24 (4)	0	30 (2)
P10	0	2 (1)	0	0	2 (<1)
P13	2 (1)	4 (2)	6 (1)	6 (2)	18 (1)
GI.4	0	0	2 (<1)	2 (1)	4 (<1)
P4	0	0	2 (<1)	2 (1)	4 (<1)
GI.5	0	0	10 (2)	0	10 (1)
P4	0	0	10 (2)	0	10 (1)
GI.6	2 (1)	7 (3)	3 (1)	1 (<1)	13 (1)
P6	0	7 (3)	2 (<1)	0	9 (1)
P11	2 (1)	0	1 (<1)	1 (<1)	4 (<1)
GI.7	5 (3)	0	3 (1)	1 (<1)	9 (1)
P7	5 (3)	0	3 (1)	1 (<1)	9 (1)
GI.9	1 (1)	0	0	0	1 (<1)
P9	1 (1)	0	0	0	1 (<1)
GII.1	4 (3)	4 (2)	0	0	8 (1)
P33	4 (3)	4 (2)	0	0	8 (1)
GII.2	18 (13)	34 (14)	79 (13)	18 (5)	149 (11)
P2	0	0	1 (<1)	0	1 (<1)
P16	18 (13)	34 (14)	78 (13)	18 (5)	148 (11)
GII.3	9 (6)	43 (18)	77 (13)	61 (18)	190 (14)
P12	7 (5)	41 (17)	38 (6)	60 (17)	146 (11)
P16	1 (1)	2 (1)	15 (3	0	18 (1)
P21	1 (1)	0	19 (3)	1 (<1)	21 (2)
P30	0	0	4 (1)	0	4 (<1)
PNA3	0	0	1 (<1)	0	1 (<1)
GII.4 Hong Kong	8 (6)	1 (<1)	0	0	9 (1)
P31	8 (6)	1 (<1)	0	0	9 (1)
GII.4 Sydney	68 (47)	92 (39)	314 (52)	213 (62)	687 (52)
P4	8 (6)	0	0	0	8 (1)
P16	53 (37)	46 (19)	225 (38)	75 (22)	399 (30)
P31	7 (5)	46 (19)	89 (15)	138 (40)	280 (21)
GII.4 untypable	3 (2)	2 (1)	26 (4)	5 (1)	36 (3)
P4	3 (2)	2 (1)	26 (4)	2 (1)	33 (2)
P31	0	0	0	3 (1)	3 (<1)
GII.6	10 (7)	21 (9)	17 (3)	16 (5)	64 (5)
P7	10 (7)	21 (9)	17 (3)	16 (5)	64 (5)
GII.7	5 (3)	5 (2)	2 (<1)	3 (1)	15 (1)
P7	5 (3)	5 (2)	2 (<1)	3 (1)	15 (1)
GII.8	0	0	1 (<1)	0	1 (<1)
P8	0	0	1 (<1)	0	1 (<1)
GII.12	0	7 (3)	23 (4)	6 (2)	36 (3)
P16	0	7 (3)	23 (4)	6 (2)	36 (3)
GII.13	5 (3)	3 (1)	2 (<1)	7 (2)	17 (1)
P16	3 (2)	3 (1)	1 (<1)	7 (2)	14 (1)

Genotype, P-type	Season, no. (%)				Total
	2016–2017	2017–2018	2018–2019	2019–2020	no. (%)
P21	2 (1)	0	1 (<1)	0	3 (<1)
GII.14	1 (1)	1 (<1)	4 (1)	1 (<1)	7 (1)
P7	1 (1)	1 (<1)	4 (1)	1 (<1)	7 (1)
GII.17	0	6 (3)	7 (1)	0	13 (1)
P17	0	5 (2)	6 (1)	0	11 (1)
P31	0	1 (<1)	1 (<1)	0	2 (<1)
GII.20	0	0	0	2 (1)	2 (<1)
P7	0	0	0	1 (<1)	1 (<1)
P20	0	0	0	1 (<1)	1 (<1)
Total	144 (100)	238 (100)	600 (100)	343 (100)	1,325 (100)

Appendix Table 2. Dual-typed norovirus sequences from children, Australia, 2016–2020

Dual type	Season, no. (%)				Total, no. (%)
	2016–2017	2017–2018	2018–2019	2019–2020	
GII.4 Sydney[P16]	3 (16)	3 (16)	12 (43)	2 (40)	20 (28)
GII.2[P16]	3 (16)	1 (5)	9 (32)	0	13 (18)
GII.3[P12]	0	5 (26)	3 (11)	2 (40)	10 (14)
GII.6[P7]	1 (5)	6 (32)	0	0	7 (10)
GII.4 Sydney[P31]	3 (16)	1 (5)	2 (7)	0	6 (8)
GII.4 Sydney[P4]	5 (26)	0	0	0	5 (7)
GII.4 untypable[P4]	2 (11)	0	0	1 (20)	3 (4)
GI.2[P2]	0	2 (11)	0	0	2 (3)
GII.7[P7]	1 (5)	1 (5)	0	0	2 (3)
GI.6[P11]	1 (5)	0	0	0	1 (1)
GI.7[P7]	0	0	1 (4)	0	1 (1)
GII.14[P7]	0	0	1 (4)	0	1 (1)
Total	19 (100)	19 (100)	28 (100)	5 (100)	71 (100)

Appendix Table 3. Dual-typed norovirus sequences from children, Bangladesh, 2018–2020

Dual type	Season, no. (%)		Total, no. (%)
	2018–2019	2019–2020	
GII.4 Sydney[P16]	11 (39)	4 (100)	15 (47)
GII.3[P16]	12 (43)	0	12 (38)
GI.3[P13]	1 (4)	0	1 (3)
GI.3[P3]	1 (4)	0	1 (3)
GII.2[P2]	1 (4)	0	1 (3)
GII.4 Sydney[P31]	1 (4)	0	1 (3)
GII.6[P7]	1 (4)	0	1 (3)
Total	28 (100)	4 (100)	32 (100)

Appendix Table 4. Dual-typed norovirus sequences from children, Brazil, 2018–2019

Dual type	2018–2019, no. (%)
GII.4 Sydney[P16]	13 (93)
GII.6[P7]	1 (7)
Total	14 (100)

Appendix Table 5. Dual-typed norovirus sequences from children, Canada, 2018–2019

Dual type	2018–2019, no. (%)
GII.4 Sydney[P16]	55 (61)
GII.12[P16]	20 (22)
GII.3[P12]	5 (6)
GII.2[P16]	4 (4)
GII.4 Sydney[P31]	2 (2)
GI.3[P13]	1 (1)
GI.3[P3]	1 (1)
GI.7[P7]	1 (1)
GII.3[P30]	1 (1)
Total	90 (100)

Appendix Table 6. Dual-typed norovirus sequences from children, Chile, 2018–2019

Dual type	2018–2019, no. (%)
GII.4 untypable[P4]	23 (53)
GII.4 Sydney[P16]	18 (42)
GI.3[P3]	1 (2)
GII.17[P31]	1 (2)
Total	43 (100)

Appendix Table 7. Dual-typed norovirus sequences from children, Germany, 2018–2020

Dual type	Season, no. (%)		Total, no. (%)
	2018–2019	2019–2020	
GII.4 Sydney[P16]	21 (32)	10 (22)	31 (28)
GII.3[P21]	19 (29)	1 (2)	20 (18)
GII.4 Sydney[P31]	6 (9)	11 (24)	17 (15)
GII.3[P12]	1 (2)	11 (24)	12 (11)
GII.2[P16]	10 (15)	1 (2)	11 (10)
GII.6[P7]	3 (5)	7 (15)	10 (9)
GII.12[P16]	0	2 (4)	2 (2)
GI.3[P13]	0	1 (2)	1 (1)
GI.3[P3]	1 (2)	0	1 (1)
GI.4[P4]	1 (2)	0	1 (1)
GI.6[P11]	0	1 (2)	1 (1)
GI.6[P6]	1 (2)	0	1 (1)
GII.13[P16]	1 (2)	0	1 (1)
GII.4 untypable[P4]	1 (2)	0	1 (1)
GII.7[P7]	0	1 (2)	1 (1)
Total	65 (100)	46 (100)	111 (100)

Appendix Table 8. Dual-typed norovirus sequences from children, Hong Kong, 2017–2020

Dual type	Season, no. (%)			Total, no. (%)
	2017–2018	2018–2019	2019–2020	
GII.4 Sydney[P31]	2 (10)	49 (34)	115 (72)	166 (51)
GII.4 Sydney[P16]	3 (14)	36 (25)	27 (17)	66 (20)
GII.2[P16]	7 (33)	35 (24)	8 (5)	50 (15)
GII.3[P12]	1 (5)	12 (8)	4 (3	17 (5)
GII.6[P7]	4 (19)	3 (2)	2 (1)	9 (3)
GI.3[P13]	1 (5)	2 (1)	3 (2)	6 (2)
GII.17[P17]	2 (10)	3 (2)	0	5 (2)
GII.3[P30]	0	2 (1)	0	2 (1)
GI.5[P4]	0	1 (1)	0	1 (<1)
GI.6[P11]	0	1 (1)	0	1 (<1)
GII.13[P21]	0	1 (1)	0	1 (<1)
GII.17[P31]	1 (5)	0	0	1 (<1)
GII.8[P8]	0	1 (1)	0	1 (<1)
Total	21 (100)	146 (100)	159 (100)	326 (100)

Appendix Table 9. Dual-typed norovirus sequences from children, India, 2016–2019

Dual type	Season, no. (%)			Total, no. (%)
	2016–2017	2017–2018	2018–2019	
GII.4 Sydney[P31]	2 (17)	11 (50)	0	13 (36)
GII.4 Sydney[P16]	5 (42)	5 (23)	1 (50)	11 (31)
GI.3[P13]	0	3 (14)	0	3 (8)
GII.3[P16]	0	2 (9)	1 (50)	3 (8)
GII.13[P16]	1 (8)	1 (5	0	2 (6)
GII.13[P21]	2 (17)	0	0	2 (6)
GII.6[P7]	1 (8)	0	0	1 (3)
GII.7[P7]	1 (8)	0	0	1 (3)
Total	12 (100)	22 (100)	2 (100)	36 (100)

Appendix Table 10. Dual-typed norovirus sequences from children, Japan, 2016–2019

Dual type	Season, no. (%)			Total, no. (%)
	2016–2017	2017–2018	2018–2019	
GII.4 Sydney[P31]	1 (12)	22 (59)	15 (34)	38 (43)
GII.2[P16]	5 (63)	6 (16)	9 (20)	20 (22)
GII.6[P7]	1 (12)	6 (16)	5 (11)	12 (13)
GII.4 Sydney[P16]	1 (12)	1 (3)	8 (18)	10 (11)
GII.3[P12]	0	2 (5)	7 (16)	9 (10)
Total	8 (100)	37 (100)	44 (100)	89 (100)

Appendix Table 11. Dual-typed norovirus sequences from children, New Zealand, 2018–2020

Dual type	Season, no. (%)		Total, no. (%)
	2018–2019	2019–2020	
GII.3[P12]	0	26 (67)	26 (48)
GII.4 Sydney[P31]	3 (20)	5 (13)	8 (15)
GII.4 Sydney[P16]	5 (33)	1 (3)	6 (11)
GII.2[P16]	2 (13)	2 (5)	4 (7)
GII.6[P7]	1 (7)	2 (5)	3 (6)
GI.4[P4]	1 (7)	0	1 (2)
GII.12[P16]	0	1 (3)	1 (2)
GII.20[P20]	0	1 (3)	1 (2)
GII.20[P7]	0	1 (3)	1 (2)
GII.3[P16]	1 (7)	0	1 (2)
GII.4 untypable[P4]	1 (7)	0	1 (2)
GII.7[P7]	1 (7)	0	1 (2)
Total	15 (100)	39 (100)	54 (100)

Appendix Table 12. Dual-typed norovirus sequences from children, Nicaragua, 2017–2019

Dual type	Season, no. (%)		Total, no. (%)
	2017–2018	2018–2019	
GII.4 Sydney[P16]	2 (11)	28 (47)	30 (38)
GI.3[P3]	0	16 (27)	16 (21)
GI.5[P4]	0	9 (15)	9 (12)
GII.4 Sydney[P31]	8 (42)	0	8 (10)
GII.12[P16]	7 (37)	0	7 (9)
GII.14[P7]	1 (5)	2 (3)	3 (4)
GII.17[P17]	0	3 (5)	3 (4)
GI.7[P7]	0	1 (2)	1 (1)
GII.1[P33]	1 (5)	0	1 (1)
Total	19 (100)	59 (100)	78 (100)

Appendix Table 13. Dual-typed norovirus sequences from children, Philippines, 2016–2020

Dual type	Season, no. (%)				Total, no. (%)
	2016–2017	2017–2018	2018–2019	2019–2020	
GII.4 Sydney[P16]	15 (30)	25 (42)	3 (15)	1 (33)	44 (33)
GII.2[P16]	7 (14)	11 (19)	4 (20)	1 (33)	23 (17)
GII.3[P12]	5 (10)	4 (7)	10 (50)	0	19 (14)
GII.4 Hong Kong[P31]	8 (16)	1 (2)	0	0	9 (7)
GII.6[P7]	5 (10)	3 (5)	0	1 (33)	9 (7)
GII.7[P7]	3 (6)	3 (5)	0	0	6 (5)
GI.3[P3]	1 (2)	3 (5)	0	0	4 (3)
GII.13[P16]	2 (4)	2 (3)	0	0	4 (3)
GII.17[P17]	0	3 (5)	0	0	3 (2)
GI.3[P10]	0	2 (3)	0	0	2 (2)
GI.6[P6]	0	1 (2)	1 (5)	0	2 (2)
GI.1[P1]	1 (2)	0	0	0	1 (1)
GI.3[P13]	0	0	1 (5)	0	1 (1)
GI.6[P11]	1 (2)	0	0	0	1 (1)
GI.9[P9]	1 (2)	0	0	0	1 (1)
GII.14[P7]	0	0	1 (5)	0	1 (1)
GII.4 Sydney[P31]	0	1 (2)	0	0	1 (1)
GII.4 Sydney[P4]	1 (2)	0	0	0	1 (1)
Total	50 (100)	59 (100)	20 (100)	3 (100)	132 (100)

Appendix Table 14. Dual-typed norovirus sequences from children, South Africa, 2017–2019

Dual type	Season, no. (%)		Total, no. (%)
	2017–2018	2018–2019	
GII.4 Sydney[P31]	1 (100)	10 (83)	11 (85)
GII.2[P16]	0	1 (8)	1 (8)
GII.3[PNA3]	0	1 (8)	1 (8)
Total	1 (100)	12 (100)	13 (100)

Appendix Table 15. Dual-typed norovirus sequences from children, Spain, 2018–2020

Dual type	Season, no. (%)		Total, no. (%)
	2018–2019	2019–2020	
GII.4 Sydney[P16]	4 (25)	15 (54)	19 (43)
GII.2[P16]	4 (25)	1 (4)	5 (11)
GII.4 untypable[P31]	0	3 (11)	3 (7)
GII.6[P7]	1 (6)	2 (7)	3 (7)
GI.3[P3]	2 (13)	0	2 (4)
GII.4 Sydney[P31]	0	2 (7)	2 (4)
GI.1[P1]	0	1 (4)	1 (2)
GI.3[P13]	1 (6)	0	1 (2)
GI.7[P7]	0	1 (4)	1 (2)
GII.12[P16]	1 (6)	0	1 (2)
GII.13[P16]	0	1 (4)	1 (2)
GII.14[P7]	0	1 (4)	1 (2)
GII.3[P12]	0	1 (4)	1 (2)
GII.3[P16]	1 (6)	0	1 (2)
GII.3[P30]	1 (6)	0	1 (2)
GII.4 untypable[P4]	1 (6)	0	1 (2)
Total	16 (100)	28 (100)	44 (100)

Appendix Table 16. Dual-typed norovirus sequences from children, Taiwan, 2019–2020

Dual type	2019–2020, no. (%)
GII.3[P12]	7 (37)
GII.4 Sydney[P31]	5 (26)
GII.2[P16]	3 (16)
GI.3[P13]	2 (11)
GI.4[P4]	2 (11)
Total	19 (100)

Appendix Table 17. Dual-typed norovirus sequences from children, United States, 2016–2020

Dual type	Season, no. (%)				Total, no. (%)
	2016–2017	2017–2018	2018–2019	2019–2020	
GII.4 Sydney[P16]	29 (53)	7 (12)	10 (56)	15 (38)	61 (35)
GII.3[P12]	2 (4)	29 (48)	0	9 (23)	40 (23)
GII.2[P16]	3 (5)	9 (15)	0	2 (5)	14 (8)
GII.6[P7]	2 (4)	2 (3)	2 (11)	2 (5)	8 (5)
GII.1[P33]	4 (7)	3 (5)	0	0	7 (4)
GI.6[P6]	0	6 (10)	0	0	6 (3)
GII.13[P16]	0	0	0	6 (15)	6 (3)
GI.7[P7]	5 (9)	0	0	0	5 (3)
GII.12[P16]	0	0	2 (11)	3 (8)	5 (3)
GI.3[P3]	1 (2)	1 (2)	2 (11)	0	4 (2)
GII.4 untypable[P4]	1 (2)	2 (3)	0	1 (3)	4 (2)
GII.7[P7]	0	1 (2)	1 (6)	2 (5)	4 (2)
GI.3[P13]	2 (4)	0	0	0	2 (1)
GII.4 Sydney[P31]	1 (2)	0	1 (6)	0	2 (1)
GII.4 Sydney[P4]	2 (4)	0	0	0	2 (1)
GII.14[P7]	1 (2)	0	0	0	1 (1)
GII.3[P16]	1 (2)	0	0	0	1 (1)
GII.3[P21]	1 (2)	0	0	0	1 (1)
Total	55 (100)	60 (100)	18 (100)	40 (100)	173 (100)
